# An in Vitro Study on the Effect of Combined Treatment with Photodynamic and Chemical Therapies on *Candida albicans*

**DOI:** 10.3390/ijms19020337

**Published:** 2018-01-24

**Authors:** Yi-Hsuan Hsieh, Jun-Hui Zhang, Wen-Ching Chuang, Kun-Hua Yu, Xian-Bin Huang, Yao-Chang Lee, Cheng-I Lee

**Affiliations:** 1 Department of Clinical Pathology, Buddhist Dalin Tzu Chi General Hospital, Chia-Yi 62247, Taiwan; dm989587@tzuchi.com.tw; 2 Department of Biomedical Sciences, National Chung Cheng University, Min-Hsiung, Chia-Yi 62102, Taiwan; s0994757@gmail.com (J.–H.Z.); danny7910791@gmail.com (W.–C.C.); ykhuna@gmail.com (K.-H.Y.); loveghate43@gmail.com (X.–B.H.); 3 National Synchrotron Radiation Research Center, Hsinchu 30076, Taiwan; yclee@nsrrc.org.tw

**Keywords:** photodynamic therapy, *Candida albicans*, curcumin, fluconazole, biofilm

## Abstract

*Candida albicans* is the most commonly encountered human fungal pathogen, and it is traditionally treated with antimicrobial chemical agents. The antimicrobial effect of these agents is largely weakened by drug resistance and biofilm-associated virulence. Enhancement of the antimicrobial activity of existing agents is needed for effective candidiasis treatment. Our aim was to develop a therapy that combined biofilm disruption with existing antimicrobial agents. Photodynamic therapy (PDT) utilizing curcumin and blue light was tested as an independent therapy and in combination with fluconazole treatment. Viability assays and morphology analysis were used to assess the effectiveness of *C. albicans* treatment. Results showed that fluconazole treatment decreased the viability of planktonic *C. albicans*, but the decrease was not as pronounced in adherent *C. albicans* because its biofilm form was markedly more resistant to the antimicrobiotic. PDT effectively eradicated *C. albicans* biofilms, and when combined with fluconazole, PDT significantly inhibited *C. albicans* to a greater extent. This study suggests that the addition of PDT to fluconazole to treat *C. albicans* infection enhances its effectiveness and can potentially be used clinically.

## 1. Introduction

Photodynamic therapy (PDT) is a minimally invasive therapy approved for the treatment of cancers [1] and other diseases [2]. PDT requires light at a specific wavelength to raise the electrons in photosensitizer (PS) molecules to the excited singlet state. The excited PS may cross to the triplet state—with slightly lower energy—by undergoing an intersystem crossing process. Further photochemical reaction runs along the type I or type II pathway. Along the former pathway, the triplet state PS transfers electrons to nearby molecules to produce free radicals. Along the latter pathway, it transfers energy to nearby ground-state (triplet-state) molecular oxygen to produce excited oxygen in the singlet state (singlet oxygen; ^1^O_2_). Both free radicals and singlet oxygen generated through the type I and type II pathways are cytotoxic.

*Candida albicans* is the most commonly encountered human fungal pathogen [3], and is an important opportunistic pathogen—especially in intensive care units [4] and oral infections [5]. *C. albicans* is a polymorphic fungus growing either as a unicellular budding yeast or in filamentous form including pseudohyphae and true hyphae. Pseudohyphal growth is a transition between budding and hyphal growth, and the transition is essential to virulence [6]. Traditionally, *C. albicans* infection is treated with antifungal agents such as intravenous amphotericin B, oral fluconazole, and topical clotrimazole [7]. However, the effect of antifungal agents is greatly weakened by the drug resistance ascribed to hyphal and pseudohyphal secretion of extracellular matrix from biofilms [8,9]. Moreover, fluconazole has various side effects [10,11,12,13]. Hence, alternative treatments against candidiasis are being considered [14]. For the easy access of visible light to the oral cavity, phototherapies can be applied to oral candidiasis, such as burning mouth syndrome [15,16]. From the small scale of clinical trials, phototherapy seemed to be effective in decreasing plaque size, but complete eradication of fungal pathogens is expected. In the past, antimicrobial PDTs [2,17] have included rose bengal PDT [18] and 5-aminolevulinic acid (5-ALA) PDT [19] against *C. albicans*. However, micromolar concentrations of rose bengal can cause damage, for example, to the living human corneal epithelium [20]. In humans, 5-ALA is converted to protoporphyrin IX, the photosensitizer. Typically, millimolar concentrations of 5-ALA are required to be effective. To avoid toxicity of photosensitizers and treat candidiasis efficiently, further development of anti-microbial PDTs and multi-antibiotic approaches are required.

Curcumin is a well-known antimicrobial polyphenolic compound derived from *Curcuma longa* [21]. In this study, we utilized curcumin as an antifungal photosensitizer. As biofilm formation is an important factor in the pathogenesis of *C. albicans* infection [22], our aim was to determine whether PDT combined with traditional antifungal agents could improve their antimicrobial effect against adherent *C. albicans*.

## 2. Results

### 2.1. Generation of Free Radicals and Singlet Oxygen

**Figure 1:**
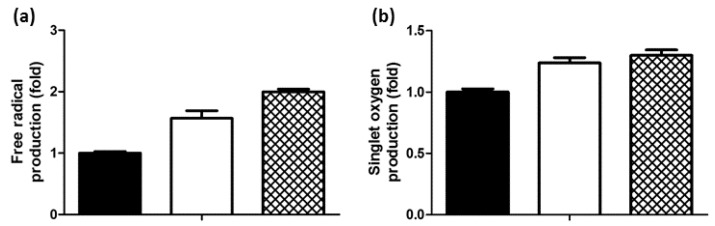
Generation of (**a**) free radicals and (**b**) singlet oxygen from curcumin-photodynamic therapy (PDT) detected with 1,1-diphenyl-2-picrylhydrazyl (DPPH) and 1,3-diphenylisobenzofuran (DPBF), respectively. The filled and open columns represent the untreated (control) and curcumin-PDT samples, respectively. The mesh columns represent the positive control: ascorbic acid in (**a**) and PDT with rose bengal in (**b**).

The formation of free radicals and singlet oxygen are the end products of PDT activity. Hence, the production of free radicals by curcumin-PDT was tested primarily by using a scavenger: 1,1-diphenyl-2-picrylhydrazyl (DPPH). As shown in Figure 1a, upon illumination of curcumin, free radicals were generated at about 82% of the levels scavenged by ascorbic acid—an outstanding antioxidant used here as a positive control. We used 1,3-diphenylisobenzofuran (DPBF) to assay the amount of singlet oxygen formation. As shown in Figure 1b, the amount of singlet oxygen produced by illumination of curcumin was comparable to that produced by the illumination of rose bengal (a well-known photosensitizer and efficient producer of singlet oxygen [23] used here as a positive control).

### 2.2. Viability of C. albicans after Treatment with Antifungal Agent

**Figure 2:**
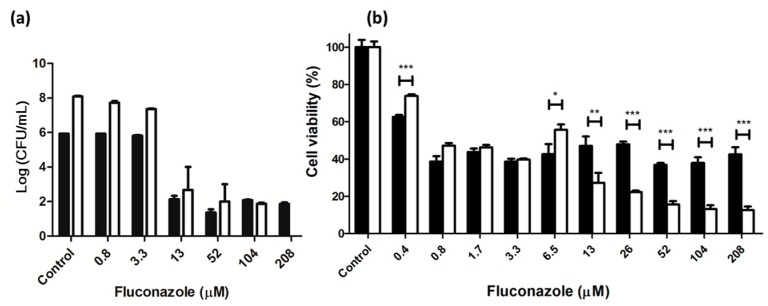
Viability of *C. albicans* in (**a**) planktonic culture and (**b**) adherent culture after treatment with fluconazole. The filled and unfilled columns represent treatments for 24 h and 48 h, respectively. * *p* < 0.05, ** *p* < 0.01, *** *p* < 0.001.

The effect of fluconazole—a well-known antifungal agent—on colony formation and viability of *C. albicans* in suspension is shown in Figure 2. Fluconazole had antifungal activity at 13 μM or higher, and antifungal activity was maximal at 13 μM after 24 h of treatment. At 48 h of treatment, no colonies of *C. albicans* were observed in the presence of 208 μM fluconazole. The viability of adherent *C. albicans* was about 40% even at 208 μM after 24 h of treatment and ~15% at 208 μM after 48 h of treatment. Moreover, dose dependence was pronounced at higher than 13 μM after 48 h of treatment. Clearly, anticandidal activity of fluconazole is great in *C. albicans* suspension after prolonged treatment, but greatly weakened by the formation of adherent *C. albicans* biofilms. The weakening of antifungal activity by biofilm formation should be considered in further PDT experiments.

### 2.3. PDT Effect on Planktonic and Adherent C. albicans

**Figure 3:**
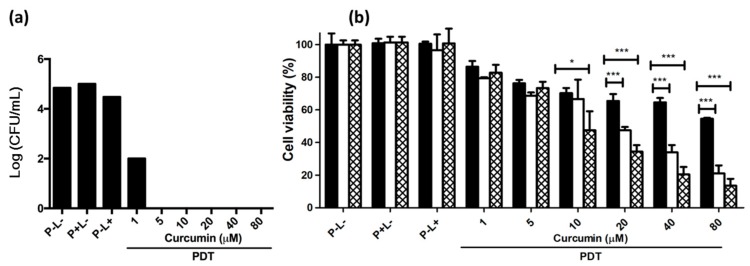
Viability of *C. albicans* in (**a**) planktonic culture and (**b**) adherent culture after PDT treatment. The total illumination time was 30 min. In the experiment of adherent *C. albicans*, illumination was carried out in one (filled), three (unfilled), or six (mesh) cycles. P and L represent photosensitizer and light, respectively. The symbol + indicates that the specific factor was used, and—indicates that the specific factor was not used. * *p* < 0.05, *** *p* < 0.001.

*C. albicans* grown in both planktonic and adherent cultures was exposed to PDT. As shown in Figure 3a, PDT utilizing 1 μM curcumin and 9 J/cm^2^ of light was sufficient to decrease the number of planktonic *C. albicans* colonies by three orders of magnitude. At curcumin concentrations of 5 μM or higher, PDT eliminated all *C. albicans* colonies. Therefore, PDT is very effective when *C. albicans* is in suspension. This result is very similar to findings from a previous study [24], which used a greater light dose.

*C. albicans* in biofilms was more drug-resistant and virulent. As dispersion is an important step in the development of *C. albicans* biofilms, and biofilm formation can greatly reduce the effectiveness of antifungal agents [22,25], we exposed adherent *C. albicans* to PDT. Figure 3b shows that the decrease in cell viability after continuous PDT illumination for 30 min (9 J/cm^2^) was weak and less in adherent *C. albicans* than in planktonic *C. albicans*. Intermittent PDT illumination (3–6 periods of illumination over a total of 30 min) greatly enhanced the effect, and significantly decreased cell viability. This improvement should be ascribed to the rapid photobleaching of curcumin [24]. PDT utilizing 80 μM curcumin and six periods of illumination reduced cell viability to ~15%. However, good antifungal treatment requires the development of a more efficient therapy that totally prevents replication.

### 2.4. The Effect of Chemical Therapy Combined with PDT on C. albicans in Adherent Culture

**Figure 4:**
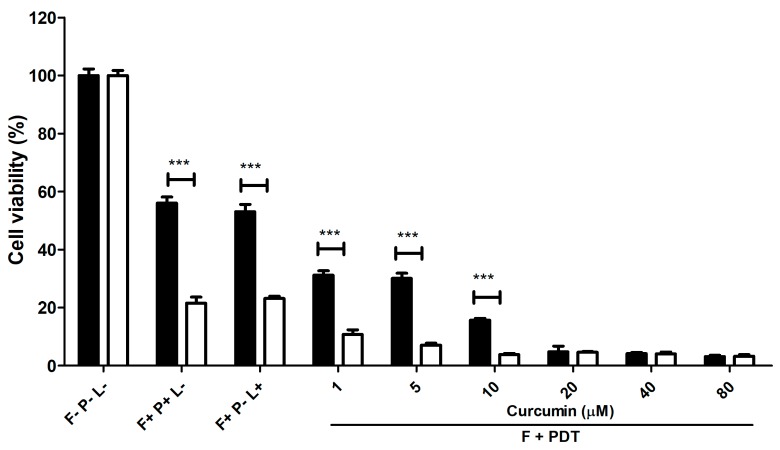
Viability of *C. albicans* in adherent culture after sequential treatment with fluconazole for 24 h (filled) or 48 h (open) and PDT utilizing curcumin. F, P, and L represent fluconazole, photosensitizer, and light, respectively. The symbol + indicates that the specific factor was used, and—indicates that the specific factor was not used. *** *p* < 0.001.

Neither PDT nor fluconazole treatment is efficient enough to eliminate *C. albicans* completely in adherent culture. Therefore, sequential treatments with 208 μM of fluconazole and PDT utilizing various concentrations of curcumin were tested. As shown in Figure 4, treatment with fluconazole for 24 h and 48 h decreased the cell viability to 55% and 20%, respectively. Fluconazole treatment for 24 h followed by PDT utilizing 20 μM curcumin (or fluconazole-treatment for 48 h followed by PDT utilizing 10 μM curcumin) decreased cell viability to 5%. Obviously, the combination of chemical therapy and PDT greatly promotes the inactivation of *C. albicans*.

### 2.5. Cellular Morphology of C. albicans before and after PDT or Chemical Therapy or Both

**Figure 5:**
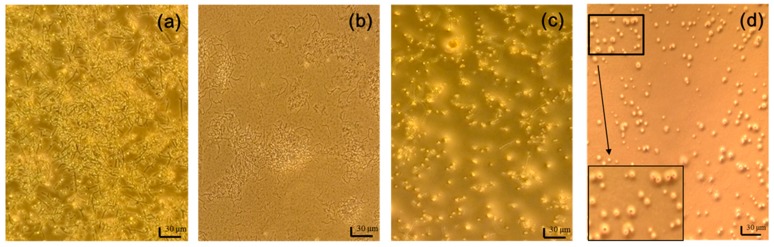
Light images of *C. albicans* (**a**) before the treatment, and after the treatments with (**b**) 208 μM fluconazole; (**c**) PDT; and (**d**) combination of fluconazole and PDT. The scale bar represents 30 μm. The surface features in the black box are enlarged for better visualization.

**Figure 6:**
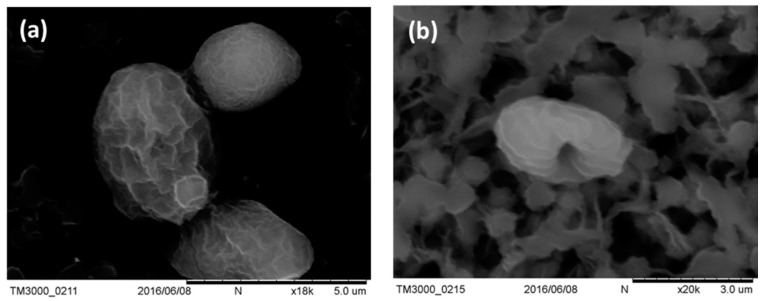
SEM images of *C. albicans* (**a**) before and (**b**) after PDT treatments.

Adherent *C. albicans* cells were yeast-like with long pseudohyphae, and formed a biofilm as shown in Figure 5a. Treatment with 208 μM fluconazole significantly decreased the presence of yeast-like cells, but not pseudohyphae (Figure 5b). In contrast, PDT removed most of the biofilm mass, but left some yeast-like cells (Figure 5c). The extent of biofilm damage by curcumin-PDT was similar to that by gold nanoparticles (AuNP) treatment [26]. After fluconazole combined with PDT, few yeast-like cells remained and the biofilm was completely eliminated. Most of these yeast-like cells lacked some surface features, as shown in the insert of Figure 5d. Further examination by scanning electron microscope (SEM) (Figure 6) showed that morphological change in *C. albicans* after our combination treatment was similar to that after porphyrin PDT as reported previously [27]. These images demonstrate that chemical therapy and PDT can truly eradicate *C. albicans* cells and disrupt biofilms.

### 2.6. PDT-Induced Peroxidation of Membranes

**Figure 7:**
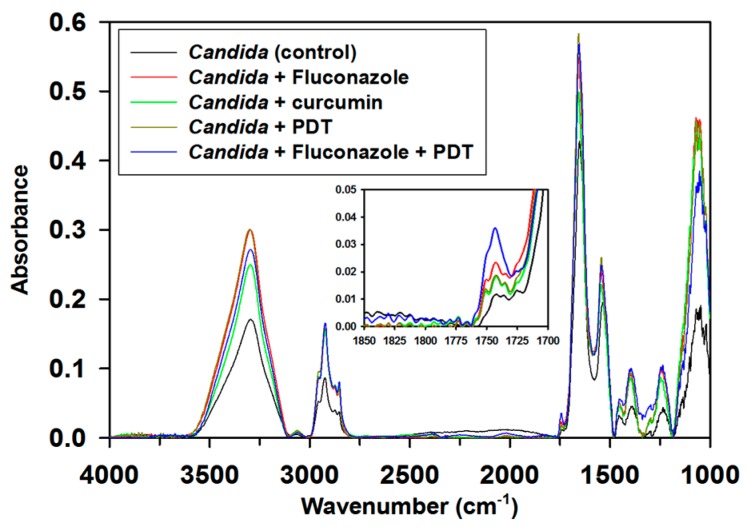
Fourier transform infrared (FT-IR) spectra of *C. albicans* before the treatment and after the treatments with fluconazole, curcumin, PDT, and combination of fluconazole therapy and PDT.

Fourier transform infrared (FT-IR) spectra (Figure 7) identified the vibrational features of proteins (i.e., amide I, amide II, and amide III at 1900–1600 cm^–1^, 1600–1500 cm^–1^, and 1400–1300 cm^–1^, respectively), phospholipids (CH_2_ stretch at ~2900 cm^–1^, ester C=O stretch at 1740 cm^–1^, phosphodiester asymmetric stretch at 1250 cm^–1^, and phosphodiester symmetric stretch at 1085 cm^–1^), and cell wall polysaccharides (1200–900 cm^–1^). The band at 1740 cm^–1^ indicating the ester functional groups of the phospholipids is regarded as an index band for the peroxidation of phospholipids [28]. Sequential treatment with fluconazole and PDT (in contrast to other treatments) resulted in marked increase in the intensity of this index band, as shown in the insert of Figure 7. This result suggests that combination therapy further enhances the peroxidation of phospholipids in *C. albicans*. The peroxidation of phospholipids might correlate with the change in the surface morphology of *C. albicans* cells.

## 3. Discussion

Since the early 1980s, fungal infections have been a major problem all over the world, especially in immunocompromised individuals [29] and oral cavities [30]. Alternative therapies have been suggested [14]. One of these, curcumin, has been considered a promising anticandidal drug [31] because of its inhibitory activity and preventive activity on the development of *C. albicans* biofilms on denture surfaces [32]. Curcumin is effective at sub-millimolar concentration. However, the bioavailability of curcumin is compromised by poor solubility. The healing power of curcumin can be enhanced by increasing its solubility through heating [33], dispersion [34], nanoparticle fabrication [35,36], and metal chelation [37]. In the current work, only micromolar amounts of curcumin are needed, so solubility is not a problem.

For the easy access of light to the oral cavity, photo-associated therapies have been applied to oral fungal infection [15,16]. To further eradicate the fungal pathogens, PDT has been considered [2,17]. For the efficacy of PDT, PDT has been clinically applied to oral cancer and some other cancers [38]. However, the difficulty of antifungal therapy is to overcome biofilms which are critical in virulence and drug-resistance.

Because of the need for new improved antifungal therapies, several combination antifungal therapies have been proposed [39,40]. Combining antifungal drugs was recommended to enhance the efficiency of treatments for a variety of infections. However, synergistic benefits have been difficult to achieve due to losses of antifungal activity and clinical efficiency [41]. Moreover, a clinical study of combined antifungal therapies in severely immunocompromised patients found that the mortality rate for invasive fungal infections remains high [42].

Rather than combining two antifungal chemical agents, we combined two antifungal therapies with different mechanisms of action (a chemical therapy and PDT). More than two decades ago, a study found that the phototoxicity of curcumin depends on hydrogen peroxide rather than singlet oxygen [43]. A later study suggested that the curcumin phototoxicity mechanism involves both type I (O_2_ and H_2_O_2_ formation) and type II (singlet oxygen) reactions [44]. In the present study, the generation of free radicals and singlet oxygen were detected by the DPPH and DPBF assays, respectively. DPPH can trap various radicals including singlet oxygen, while DPBF specifically identifies singlet oxygen. Similar to most PDT mechanisms [1], the mechanism of our PDT against *C. albicans* should rely on singlet oxygen as the major reactive oxygen species (ROS), but we cannot rule out the toxicity of other radical species.

PDT has been used against a variety of microbial pathogens [45]. In a previous study, antimicrobial PSs were synthesized to carried out PDT under high illumination energy (40 J/cm^2^), but the effect on the biofilms was not identified [46]. Two studies have reported the effectiveness of a PDT with 40 μM curcumin and blue light against *C. albicans* [24,47]. In a murine model, PDT utilizing 80 μM of curcumin and 37.5 J/cm^2^ of blue light significantly decreased the viability of *C. albicans* [44]. Combined treatment with photodynamic and chemical therapies has been applied to cancer treatments [48,49], but has not been applied to fungal infections. In the current work, fluconazole was a very efficient inhibitor of *C. albicans* replication. However, virulence caused by biofilm formation made eradicating biofilms an important issue. In our study, 5 μM curcumin and 9 J/cm^2^ of blue light were sufficient to eradicate biofilms. Thus, combining fluconazole with PDT can both inhibit *C. albicans* replication and eradicate biofilms completely. Notably, the eradication of biofilms is to completely inhibit the virulence of the fungal pathogens and to prevent drug resistance in the chemical therapy.

## 4. Materials and Methods

### 4.1. Measurement of Free Radicals and ^1^O_2_ Generation

The amount of free radicals generated after illumination in the presence of curcumin was determined based on the scavenging activity of DPPH. Equal volumes of 25 μM DPPH and 0.5 μM of curcumin were mixed in ethanol solution and subsequently illuminated to activate the redox reaction (see PDT experiments for details). In the positive control, curcumin was replaced by an equal concentration of ascorbic acid (no illumination). After 30 min of scavenging reaction, the decrease in DPPH absorbance was measured at 517 nm.

The amount of singlet oxygen generated after illumination was determined by DPBF. Equal volumes of 10 μM DPBF and 1 μM curcumin mixed in ethanol solution were used for fluorescence measurements and PDT experiments. The excitation and monitored emission wavelengths were 403 nm and 450 nm, respectively. Comparatively, rose bengal—a well-known photosensitizer that produces large amounts of singlet oxygen—was used as a positive control.

### 4.2. Culture of Planktonic C. albicans and Planktonic Cell Viability

*C. albicans* (ATCC 90029) suspensions (3 × 10^8^ cells/mL) were cultured in glass tubes at 37 °C. To test fluconazole as the chemical therapy, it was added to *C. albicans* in TGC medium and incubated at 37 °C for 24 h or 48 h. To determine cell viability, aliquots of 100-fold (10,000-fold dilution for the control) serial dilutions of each sample were seeded on chocolate agar plates. All plates were aerobically incubated at 37 °C for 18 h, and the number of colony forming units per milliliter (CFU/mL) was calculated from the plate counts. According to the National Committee for Clinical Laboratory Standards, the minimum inhibitory concentration of fluconazole for *C. albicans* is 208 μM (64 mg/L) [50]. In this study, the fluconazole concentration was lower than 208 μM.

### 4.3. Culture of Adherent C. albicans and Adherent Cell Viability

Primarily, 100 μL of *C. albicans* suspension was transferred into a 96-well culture plate at concentration of 3 × 10^8^ cells/mL and incubated at 37 °C in an orbital shaker at 75 rpm for 1.5 h. Subsequently, samples were washed twice with 150 μL of phosphate-buffered saline (PBS) to remove suspended cells. To allow *C. albicans* to form a biofilm, 150 μL of fresh TGC medium was added for further incubation at 37 °C for 18 h with shaking at 75 rpm. Afterwards, the samples were carefully washed twice with 200 μL of PBS to remove the remaining nonadherent cells.

The cell viability assay using water soluble tetrazolium salt WST-1 (2-(4-Iodophenyl)-3-(4-nitrophenyl)-5-(2,4-disulfophenyl)-2*H*-tetrazolium) was used to measure the viability of adherent *C. albicans* cells. The addition of 100 μL of medium containing 5 μL of WST-1 was followed by incubation for 4 h at 37 °C in the dark and measurement of formazan absorbance at 450 nm in an ELISA reader. For statistical analysis, a *t*-test was performed. The reported values are the mean values of three replicates. Statistical significance was indicated at * *p* < 0.05, ** *p* < 0.01, or *** *p* < 0.001, depending on the experiments.

### 4.4. PDT Experiments

PDT experiments were carried out by illuminating curcumin-treated *C. albicans* using a home-made device consisting of an array of 24 blue LEDs (peak emission wavelength at 430 nm). The accumulated photoenergy after 30 min of illumination was 9 J/cm^2^. Curcumin solutions were added to *C. albicans* suspensions to reach final curcumin concentrations of 1, 5, 10, 20, 40, and 80 μM. One mL of the *C. albicans*–curcumin mixture was transferred to a 12-well plate and incubated in the dark for 20 min. Subsequent illumination activated the PDT. In experiments combining PDT with chemical therapy, *C. albicans* was treated with 208 μM fluconazole prior to the PDT experiments.

### 4.5. Scanning Electron Microscopy (SEM)

*C. albicans* samples were collected on 0.45 μm filter paper, fixed with 2.5% glutaraldehyde for 1 h, dehydrated by washing with a series of ethanol solutions (25%, 50%, 75%, and 95% for 5 min, and 99.5% for 1 h), and finally dehydrated by critical point drying with carbon dioxide. After coating with gold, the samples were imaged under an electron microscope (Hitachi TM3000, Hitachi, Tokyo, Japan).

### 4.6. FT-IR Microspectroscopy of C. albicans after PDT and/or Chemical Treatment

The FT-IR spectroscopy of single cells was performed by using a FTIR spectrometer (Nicolet 6700, Thermo-Fisher Scientific, Madison, WI, USA) with 4 cm^−1^ spectral resolution and 900–3600 cm^−1^ scanning range. For each sample, 20 spectra in different fields (20 μm × 20 μm) were collected with a confocal infrared microscope (Nicolet Continuum; Thermo-Fisher Scientific, Madison, WI, USA) at infrared microspectroscopy endstation BL14A1 of National Synchrotron Research Radiation Center. Each representative spectrum is an average of 20 spectra.

## 5. Conclusions

*C. albicans* is the most common superficial fungal infection in humans [51,52]. An effective therapy to kill the cells and reduce their biofilm-associated virulence is required. Fluconazole greatly eliminates the yeast form, but not the biofilms, whereas curcumin-PDT has the opposite effect. Therefore, the combination of fluconazole treatment and curcumin-PDT to fully eradicate the growth and virulence of *C. albicans* has clinical potential. Similarly, the combination of chemical therapy and PDT could also be helpful in fighting other pathogens.

## Abbreviations

PDT	Photodynamic therapy
PS	Photosensitizer
^1^O_2_	Singlet oxygen
5-ALA	5-aminolevulinic acid
DPPH	1,1-diphenyl-2-picrylhydrazyl
DPBF	1,3-diphenylisobenzofuran
CFU	Colony forming units
SEM	Scanning electron microscopy
FT-IR	Fourier transform infrared
